# *Real-life* data of hepatitis C treatment with direct acting antiviral therapy in persons injecting drugs or on opioid substitution therapy

**DOI:** 10.1007/s15010-024-02433-4

**Published:** 2024-11-11

**Authors:** Pfaeffle M., Duenkelmann S., Boesecke C, Rockstroh J.K., Schwarze-Zander C.

**Affiliations:** 1 Gemeinschaftspraxis Am Kaiserplatz, Bonn, Germany; 2 Department of Internal Medicine, University Hospital Bonn, Bonn, Germany; 3 Deutsches Zentrum Für Infektionsforschung, Partnersite Cologne-Bonn, Germany

**Keywords:** HCV, PWID, DAA, Adherence

## Abstract

**Purpose:**

HCV treatment has been revolutionized by introduction of direct-acting antiviral therapy (DAA). Short treatment duration of eight to twelve weeks combined with significantly improved tolerability opened the opportunity to reach out to difficult-to-treat populations. Here, we retrospectively analyzed *real life* data on HCV treatment adherence and outcome in people who inject drugs (PWID) or on opioid substitution therapy (OST).

**Methods:**

All PWID or on OST receiving DAA therapy between 3/2021–11/2022 at an infectious disease clinic in Bonn were retrospectively analyzed. Patients received either 8 weeks glecaprevir/pibrentasvir or 12 weeks sofosbuvir/velpatasvir (+ ribavirin in genotype 3 cirrhotic patients). Sustained virological response (SVR) was measured 4 and 12 weeks after HCV therapy.

**Results:**

In our cohort 47 patients (68%) received treatment with glecaprevir/pibrentasvir and 22 patients (32%) sofosbuvir/velpatasvir. All 47 (100%) patients started on glecaprevir/pibrentasvir received prescriptions for the full length of therapy, while patients on sofosbuvir/velpatasvir completed 12 weeks therapy in 86% and 8 weeks in 14% (p = 0.029).

Of 69 patients 74% were found to achieve SVR. In 20% no information is available as they were lost to follow-up. Re-infection was documented in 3 patients and one relapse in a gt3 patient with cirrhosis.

**Conclusion:**

High adherence and response rates to HCV treatment were found following DAA based therapy in PWID supporting the call to include difficult-to-treat populations into HCV treatment efforts on the way to HCV elimination. Treatment of OST and HCV at one institution supporting patients by a multidisciplinary team may further facilitate adherence to follow up visits enabling documentation of treatment outcomes more easily.

## Introduction

Chronic hepatitis C virus (HCV) infection is a global health problem affecting about 71 million people worldwide with around 1.5 million new infections every year. Up to now there is no effective vaccine against hepatitis C, so reaching the WHO goal of reducing hepatitis infections between 2016 and 2030 by 90% includes scaling up HCV testing, linkage to care, retention in care, treatment of chronic HCV infection and preventing HCV primary and re-infection [1].

Compared to other European countries the prevalence of chronic HCV infection in the German population is low with 0.3% ((DEGS1). However, there are great differences between different key population groups, with people in prison reaching 14.3% and homeless people reaching 16%. The most vulnerable group for HCV infection, however, are people who inject drugs (PWID), thus being a key population when aiming at HCV elimination. Globally, 52% of PWID are HCV-antibody positive[2], a recent study in Germany found chronic HCV infection in 23–54% of PWID [3].

The advent of direct-acting antiviral agents (DAAs) has revolutionized HCV treatment and may be a game-changer on the path to HCV elimination. The usually short eight to twelve weeks therapy with neglectable side-effects and highly effective HCV eradication exceeding 95% allows treatment in difficult-to-treat populations, such as persons injecting drugs or on opioid substitution treatment. Access to HCV treatment in this marginalized populations has been improved but still remains suboptimal, as some clinicians are concerned about incompliance, drug-drug interaction and risk of reinfection in PWID.

The aim of our study was to retrospectively acquire *real life* data on HCV treatment adherence and response in PWID or on opioid substitution therapy in a German single center infectious disease clinic.

## Methods

Retrospectively all patients were analyzed, who received DAA therapy at an infectious disease clinic between 1st March 2021 and 30th November 2022. From an electronic data base, the patients’ demographic and clinical information were recorded. The elasticity of the liver was evaluated by Fibroscan^®^ analysis (^©^Echosens™, Paris, Frankreich) and was performed before initiation of HCV therapy [4]. Threshold values were categorized as ≤ 7 kPa F0-F1 fibrosis, > 7–9.5 kPa F1-F2 fibrosis, > 9.5–14.5 F3-F4 fibrosis, > 14.5 kPa F4 fibrosis.

According to current guidelines patients received either glecaprevir/pibrentasvir over 8 weeks or sofosbuvir/velpatasvir over 12 weeks (in combination with ribavirin in genotype (GT) 3 patients with cirrhosis). HCV RNA was measured at baseline, week 4 and 8 of therapy in patients receiving glecaprevir/pibrentasvir and in patients treated with sofosbuvir/velpatasvir at baseline, week 4 or 8 week and week 12. To determine sustained virological response HCV RNA was measured 4 and 12 weeks after end of therapy in all patients. SVR was defined as undetectable HCV RNA 12 weeks after end of treatment or later if the follow-up after 12 weeks was missed. SVR was marked as unknown treatment response if SVR could not be confirmed 12 weeks after end of treatment or in a subsequent follow-up.

Statistical analysis was completed by using SPSS Statistics software v. 29.0 (IBM Corp., USA). Characteristics of the patient population were analyzed by calculation of frequencies, means and medians. Chi-square and Fischer’s exact test were used where appropriate for cross-tables with significance level of 95%. Comparison of means was carried out by paired t-test. This work was performed in accordance with local institutional review board (IRB) guidelines of the University of Bonn (Nr. 267/22).

## Results

### Patients characteristics

From 1st March 2021 to 30th November 2022 69 patients received DAA therapy for chronic HCV infection. The characteristics of patients retrospectively included in this study are summarized in Table 1. The majority of patients were men (81%, n = 56), 19% (n = 13) were women and the median age was 47 yrs. (IQR(inter-quartile range):39; 53). HCV genotype distribution was as follows: gt1 46% (32/69), gt2 1% (1/69), gt3 45% (31/69) and gt4 6% (4/69) (n = 1 not determined). HIV co-infection and HBV co-infection were found in 1 patient each and 1 patient was HCV/HBV/HDV (hepatitis C/B/D) triple-infected. Fibroscan^®^ analysis was performed in 55/69 (80%) patients. In 22 patients there was no sign of significant fibrosis (F0-F1), 9 patients showed F1-F2 fibrosis, 10 patients F3-F4 and 14 patients F4 fibrosis.

**Table 1:** Characteristics of the study population

Population		
Total, *n* (%)	69	(100)
Gender, *n* (%)		
Female	13	(19)
Male	56	(81)
Diverse	0	(0)
Age in years, median [IQR]	47	[39;53]
HCV genotype, *n* (%)		
1	32	(46)
2	1	(1)
3	31	(45)
4	4	(6)
na	1	(1)
Stage of fibrosis, *n* (%)		
F0-F1	22	(32)
F1-F2	9	(13)
F3-F4	10	(15)
F4 and more	14	(20)
na	14	(20)
HAV immunity, *n* (%)		
yes	39	(57)
no	27	(39)
na	3	(4)
HBV immunity, *n* (%)		
yes, post infection	29	(42)
yes, post vaccination	15	(22)
chronic infection	2	(3)
no	21	(30)
na	2	(3)
OST, *n* (%)		
yes	60	(87)
no	9	(13)
HCV RNA baseline in U/l, median [IQR] *n* = *69*	6.8*10^5^	[1.28*10^5^;2.8*10^6^]
Bilirubin baseline in mg/dl, median [IQR] *n* = *68*	0.7	[0.5;1.0]
gGT baseline in U/l, median [IQR] *n* = *69*	80	[35;143]
GPT baseline in U/l, median [IQR] *n* = *69*	81	[47;135]
GOT baseline in U/l, median [IQR] *n* = *68*	74	[50;151]
AFP baseline in ng/ml, median [IQR] *n* = *68*	3.0	[2.1;5.6]

*HAV* Hepatitis A virus, *HBV* Hepatitis B virus, *HCV* Hepatitis C virus, *OST* Opioid substitution therapy, *gGT* Gamma glutamyl transferase, *GPT* Glutamic-pyruvic transaminase, *GOT* Glutamic-oxaloacetic transaminase, *AFP* Alpha fetoprotein

### Compliance and adherence of patients to follow up visits

**Fig. 1:**
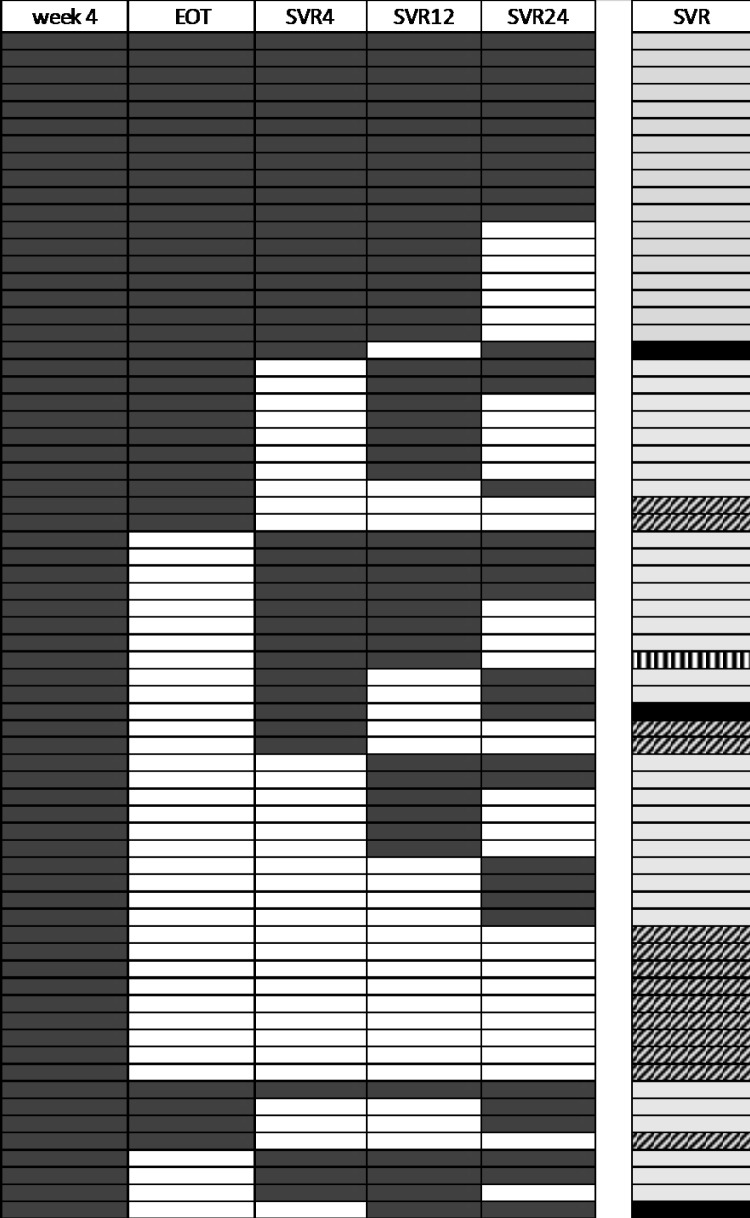
Follow-up visits of patients week 4, end of treatment (EOT), SVR 4, 12 and 24: dark grey = follow-up, white = no follow-up; SVR: light grey = successful treatment, diagonal stripes = unknown treatment response, black = reinfection, stripes = relapse

In our cohort 47 patients (68%) received treatment with glecaprevir/pibrentasvir and 22 patients (32%) sofosbuvir/velpatasvir. Figure 1 gives an overview of the follow up visits of patients on HCV therapy.

All 47 (100%) patients which started on glecaprevir/pibrentasvir came after 4 weeks of therapy and received prescriptions for the full length of an 8 weeks therapy. Patients on sofosbuvir/velpatasvir obtained the full length of 12 weeks therapy in 86% and in 14% only prescriptions for 8 weeks were collected. This difference concerning compliance in picking up the prescriptions for HCV therapy was statistically significant (p = 0.029).

In 20% (14/69) of patients follow up visits to control virological response to HCV-therapy were missed, 8/47 (17%) in the group receiving glecaprevir/pibrentasvir and 6/22 (27%) in the group receiving sofosbuvir/velpatasvir. The difference between the two HCV regimens was not significant (p = 0.3).

### Response to HCV therapy

Of the 69 patients 74% (51/69) were found to achieve SVR. 77% (36/47) after the 8 weeks therapy with glecaprevir/pibrentasvir and 68% (15/22) after the 12 weeks therapy with sofosbuvir/velpatasvir (Fig. 1). Re-infection with HCV was detected in 3 patients. In two patients reinfection was detected after SVR 12, in 1 patient reinfection was found between SVR 4 and follow up after 24 weeks, as follow up after 12 weeks was missed.

Relapse was found in only 1 patient (1%). This patient had an HCV gt 3 infection and compensated liver cirrhosis and was treated with sofosbuvir/velpatasvir plus ribavirin. However, being HCV RNA negative after 8 weeks of therapy, the patient missed the end of treatment follow up and HCV RNA was positive at the time of follow up 4 weeks after end of treatment.

In 20% (14/69) of patients no information can be given on sustained treatment response rates. Two patients had a SVR 4 virological response, but did not come back for further follow-up appointments, three patients were negative at the time of end of treatment, but did not show up for further analysis, 4 patients were negative at week 4 of therapy (2 on glecaprevir/pibrentasvir and 2 on sofosbuvir/velpatasvir), but did not come back for further analysis, 2 patients on sofosbuvir/velpatasvir were HCV RNA negative after 8 weeks of therapy, but were lost to follow-up and 1 patient on glecaprevir/pibrentasvir had 29 IU/ml, but did not come back for further follow-up appointments. Two patients had HCV viral loads of 27 IU/ml and 35 IU/ml, respectively, after 4 weeks of glecaprevir/pibrentasvir therapy, but were lost to further follow-up.

### Response of liver parameters after HCV therapy

**Fig. 2:**
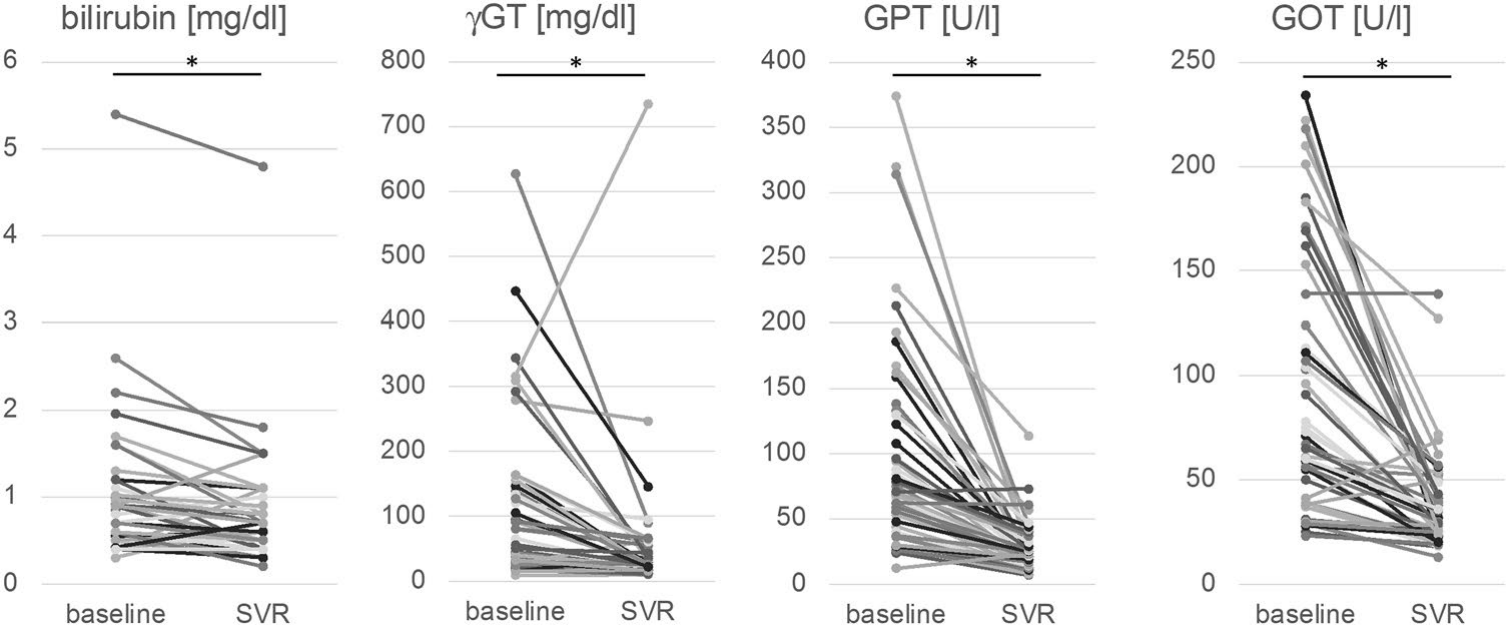
Development of bilirubin, gGT (gamma glutamyl transferase), GPT (glutamic-pyruvic transaminase) and GOT (glutamic-oxaloacetic transaminase) from baseline to SVR, *p < 0.05

The liver parameters bilirubin, gGT, GPT and GOT were measured at the beginning of HCV therapy and at SVR 12 or at a later follow up time point (Fig. 2). In the 51 patients with proven SVR the decrease of all liver parameters was significant. Bilirubin decreased from a mean value of 0.94 mg/dl to 0.76 mg/dl (p < 0.001, n = 48), gGT from a mean value of 110 U/l to 52 U/l (p = 0.001, n = 51), GPT from a mean value of 99 U/L to 27 U/l (p = 0.009, n = 51) and GOT from a mean value of 62 U/l to 25 U/l (p = 0.002, n = 49).

### Immunity against hepatitis A and B

In all individuals presenting for initiation of HCV therapy, serology for hepatitis A and B was determined.

Only 57% (39/69) showed anti-HAV antibodies and were protected against hepatitis A infection (not available n = 3). As the vaccination records were only accessible in a minority of patients, post-infection and post-vaccination against hepatitis A could not be further distinguished.

Protection against hepatitis B was detected in 64% of patients, with 22% (15/69) being anti-HBs positive after hepatitis B vaccination and 42% (29/69) showing anti-HBs and anti-HBc antibodies after past HBV infection. One patient had a chronic HBV co-infection and one patient a chronic HBV/HDV triple-infection. However, 30% (21/69) of patients had no protecting antibodies against hepatitis B with no signs of vaccination against HBV and no signs of past HBV infection.

## Discussion

In this study, we evaluated *real-life* data on the feasibility of DAA-based treatment in a single-center cohort of PWID with chronic hepatitis C. In contrast to other countries, in Germany fortunately, there are no restrictions for reimbursement of DAA therapy in PWIDs. The German national multi-centre prospective real-world registry found equally high SVR rates in patients with former or current drug use and patients never consuming drugs [5]. However, only a limited number of active drug users or individuals on substitution therapy were included. Indeed, PWID in Germany still face barriers to accessing HCV treatment, due to stigmatizing interactions with healthcare providers, discrimination in healthcare settings and limited access to healthcare due to socioeconomic factors including unstable housing [6]. In order to meet the global HCV elimination goals by 2030, Germany will have to improve the situation of PWID and prisoners substantially, mainly through the implementation of evidence-based harm reduction measures as well as the promotion of diagnosis and treatment in this particular vulnerable population [6].

Previous studies have found sustained virologic response rates following treatment with sofosbuvir-velpatasvir in 94% among PWID irrespective of drug use during therapy [7]. Here, we detected SVR in 74% of patients. However, the lower percentage found in our study does not directly reflect a lower virological response rate or higher relapse rate, but reflects the challenge of keeping a difficult-to-treat population retained in care. As much as 100% of patients receiving 8 weeks glecaprevir/pibrentasvir and 86% of patients receiving 12 weeks sofosbuvir/velpatasvir collected prescriptions for the complete time of therapy, 16% of patients on sofosbuvir/velpatasvir received prescriptions for 8 weeks of therapy. Thus, in our study a shorter duration of DAA therapy was associated with a significantly greater chance of receiving the complete DAA therapy consistent with a recent study by Martinez et al. [8]. People engaging in clinical trials reflect a population generally more engaged in health services and follow-up clinical visits are more frequent. Furthermore, clinical trials in difficult-to-treat populations provide adherence support facilities, including study nurses, pharmacists and social workers supporting retention to care [9]. In 20% of patients in our study follow-up visits were missed to analyze virological response to treatment. OST in these patients was conducted in a medical institution not treating HCV infection. Combining OST and HCV treatment in one institution with a multidisciplinary team including doctors, nurses, social workers and psychologists may increase adherence to follow-up visits substantially. Although Fibroscan^®^ analysis was available in this cohort to further characterize stage of liver fibrosis, the availability of biochemical markers is sufficient for HCV treatment decision. Also, less monitoring time points are probably necessary than requested in this study. Simplifying the initiation and monitoring of HCV therapy has recently been shown to be associated with impressive high SVR rates [10].

We detected an HCV re-infection in three patients (4%) in the first 6 months after the end of treatment. Previous studies describe an incidence of HCV reinfection of 2.7 per 100 person-years [6], however higher rates have been shown among people frequently injecting drugs [10]. Further follow-up after successful HCV therapy of PWID is needed to better understand long-term reinfection rates. Importantly, achieving HCV cure in a larger population of difficult-to-treat patients such as PWID will naturally reduce the risk of re-infection.

In the past, studies have questioned the long-term health improvement after HCV clearance in PWID. However, here we found a significant reduction of bilirubin, gGT, GPT and GOT after the course of DAA therapy. Thus, despite possible continuation of alcohol consumption or other drugs liver inflammation is declining rapidly and benefits of health improvement should focus on prevention of liver disease progression and transmission of infection. Furthermore, combining OST and HCV treatment at one institution would give a greater chance of following up patients with advanced liver fibrosis after successful HCV treatment to regularly examine for complications of liver disease such as hepatocellular carcinoma.

International guidelines recommend HAV and HBV vaccination for PWID. However, in our study 43% and 31% of patients had no immunity against hepatitis A and B, respectively. This analysis suggests that a sizeable proportion of PWID have not received HAV and/or HBV vaccination although currently recommended. Superinfection by other hepatotropic viruses, namely HAV, HBV and HBV plus HDV may lead to hepatic flares leading to liver failure in patients with chronic hepatitis C and aggravation of liver fibrosis. Low-threshold vaccination programs against hepatitis A and B need to be implemented in institutions providing social support, addiction and harm reduction programs and OST for PWID.

As this study was performed retrospectively, there are limitations concerning missed follow-up visits of patients and documentation for reasons of missed visits. Treatment adherence and response was only analyzed by measuring HCV viral load and liver enzymes. Adherence to therapy by counting missed medication or drug levels in the blood were not carried out.

In conclusion, this study demonstrates the feasibility of DAA based therapy in PWID. Barriers to access and receipt of HCV treatment for PWID need to be further reduced. Combining OST and HCV therapy at one institution supporting patients by a multidisciplinary team may increase adherence to follow up visits to enable analyzing treatment response. Given that recent PWID represent 23% of all new infections globally, HCV treatment must be increased among PWID as part of efforts to eliminate HCV globally.

## Data Availability

No datasets were generated or analysed during the current study.
